# The Preclinical Effects of Prolonged Intermittent Fasting in an Animal Model on the Development of Multiple Sclerosis Pathology

**DOI:** 10.7759/cureus.91260

**Published:** 2025-08-29

**Authors:** Drew Garber, Mohammed Pathan, Arnith Eechampati, Brandon T Smith, Jonathan Hughes, Ahad Khatri, Rohit Karnati, Hyma Muchandi

**Affiliations:** 1 Osteopathic Medicine, Philadelphia College of Osteopathic Medicine, Philadelphia, USA; 2 Neurology, River City Neurological Associates, Columbus, USA; 3 Internal Medicine, Philadelphia College of Osteopathic Medicine, Suwanee, USA; 4 Neurology, Philadelphia College of Osteopathic Medicine, Suwanee, USA; 5 Otolaryngology, Philadelphia College of Osteopathic Medicine, Suwanee, USA; 6 Anesthesiology, Philadelphia College of Osteopathic Medicine, Suwanee, USA; 7 Neurology, Mercer University School of Medicine, Macon, USA

**Keywords:** daily caloric restriction, inflammation, intermittent fasting, multiple sclerosis, neurodegeneration, nutrition and metabolism, relapse-remitting, symptomatic management of multiple sclerosis

## Abstract

Multiple sclerosis (MS) is a chronic, immune-mediated neurological disorder that affects many individuals globally, with a marked female predominance. MS is characterized by demyelination within the central nervous system (CNS), leading to impaired synaptic transmission and a wide range of neurological symptoms, including visual disturbances, motor dysfunction, cognitive decline, and vertigo. The pathophysiology of MS involves autoreactive white blood cells attacking the myelin sheath, resulting in lesion formation and progressive neuronal damage. Despite advancements in pharmacologic therapies, such as interferons, chemotherapeutic agents, and immunosuppressants like glatiramer acetate and Novantrone, current treatments primarily aim to mitigate symptoms or delay disease progression, often at the cost of severe side effects. These therapies do not address the root causes of the disease, and patient responses vary significantly. Recent research has explored novel approaches to MS management, including non-pharmacologic interventions. This paper proposes the use of prolonged intermittent fasting (IF) (>6 months) as a therapeutic strategy for MS. Emerging preclinical evidence, particularly in animal models, suggests that IF may reduce neuroinflammation, promote remyelination, and improve motor outcomes, thereby enhancing quality of life while minimizing reliance on medications with potentially life-threatening adverse effects. These findings come from the existing literature and are not derived from the present study, but they provide a rationale for further preclinical investigation. Although clinical data remains limited, this proposal highlights the need for further investigation into IF as a promising adjunct or alternative treatment modality for MS.

## Introduction and background

Multiple sclerosis (MS) is a very prevalent disease, affecting roughly 302 out of 100,000 persons in the twenty-first century, amassing nearly two-and-a-half million individuals globally. Women are three times more likely to develop MS [1]. With initial symptoms presenting between the ages of 20 and 50, this disabling neurological disease attacks the myelin layer of an axon, the outer sheath that encapsulates the conducting medium of neurons [1]. When there is damage to or a lack of a myelin sheath within the central nervous system, synaptic transmission is compromised, resulting in many underlying issues, such as blurred or double vision, short-term memory loss, muscle spasms, and vertigo [2].

MS can be considered an autoimmune condition because our immune system is destroying our axons, which aid in the conduction of electric impulses in the central nervous system (CNS). The white blood cells of our body, which are of paramount importance to our immune system, recognize the insulating layer as foreign and start attacking the myelin, causing vision loss, motor issues, and other vital processes that require functioning nerve conduction. This can lead to the development of lesions that may partially or permanently block the electrical impulse required to lift an arm or move a leg. A large area of research in academia today includes developing proper treatment and potential cures for patients with ongoing MS; the MS Research Program at the United States Department of Defense was granted $16 million for MS research alone [2].

Current MS treatments

The current research is focused on developing pharmaceutical treatments that range from anti-inflammatories, chemotherapies, and steroidal drugs, all of which try to treat or dampen the hallmark symptoms of MS. Popular drugs taken today for MS include Avonex, Betaseron, Novantrone, and many others [2]. Although these drugs seek to diminish the symptoms of MS, they ignore the underlying mechanisms and root causes of this threatening autoimmune disease. Many of the current drug regimens for MS work by reducing MRI lesion activity or by preventing secondary progressive MS, which is a worsening in the disease state. Patients have varying reactions to these drugs and do not all achieve the same ameliorating effect from the same treatments [2]. Furthermore, these drugs have minor to life-threatening side effects, ranging from allergic reactions to progressive multifocal leukoencephalopathy. For instance, the accepted first-line medication for relapsing remitting MS includes glatiramer acetate, which has noted side effects ranging from chest pain, palpitations, and throat constriction to less common side effects, including breast cancer and cutaneous lymphoma. Experimental models of MS have suggested potential for partial lesion repair and symptomatic improvement [2]. While these findings remain preclinical, advances in treatment approaches have contributed to improved quality of life for many patients and, in some cases, have reduced reliance on medications with severe adverse side effects.

Intermittent fasting as a therapeutic approach

This proposal examines prolonged intermittent fasting (IF) (>6 months) as an intervention in the experimental autoimmune encephalomyelitis (EAE) mouse model of MS. Although the benefits of caloric restriction in this context have not been experimentally tested, this paper aims to discuss how IF may influence MS-like symptoms in preclinical models, with potential implications for future human research. IF has been heavily implicated in scientific literature as a way to improve metabolism and provide cognitive benefits [3]. Much of today’s research in eating habits surrounds the quality and quantity of food and nutritional value. An equally important area of study is the time frame we choose to eat and how many hours are allocated to the intake of calories. Studies on human subjects have shown promising evidence that IF is associated with decreased systolic blood pressure and reduced low-density lipoprotein (LPL) levels [3]. Calorie restriction, and more specifically, IF has been a therapeutic approach in recent times for decreasing total body mass, improving glucose homeostasis, reducing blood cholesterol, and aiding in a variety of metabolic diseases [4]. Research also demonstrates that IF can lead to long-term cessation of overeating [5]. Obesity and its related comorbidities, including high blood pressure, high LPL levels, and insulin resistance, have been shown to contribute to a pro-inflammatory state and oxidative stress. As such, IF can lead to weight loss and A subsequent decreases in proinflammatory cytokines and other markers of immune-mediated damage. Participants in clinical trials, including obese and normal patients, are the main subjects chosen to observe the effects of IF. Still, a significant gap that needs to be filled is how different disease types are mitigated by IF, such as MS. Some variables that need to be accounted for in this study are the various time frames that can be chosen, such as the 168 or 1410 methods, where the first number represents fasting time in hours. The latter number represents the eating period, also in hours. It is critical to note which method is being adopted; if subjects choose different IF periods, the results can be significantly skewed. IF has also been shown to improve the health outcomes of patients with type 2 diabetes mellitus (T2DM) by reducing blood sugar, improving insulin resistance, and lowering body weight [6].

Key variables

A possible confounding factor of this finding includes significantly altering the diet in a patient with T2DM and how this change can cause drastic results in overall metabolism and energy expenditure. Inflammation and MS are heavily linked when looking at previous research. Moreover, the two are connected by the idea that an increase in axonal demyelination is directly proportional to a sudden increase in acute inflammation [6]. Brain inflammation and neuronal degeneration are also heavily linked, as an increase in inflammation correlates to a rise in brain lesions and other signs of MS. This is extremely important to note because if it’s known that inflammation within the brain is one of the leading causes of lesions associated with neurodegeneration in MS patients, it can be concluded that a treatment that aggressively attacks the inflammatory side of things for MS can have a sizable relief in axonal demyelination and thoroughly relieve some of the hallmark symptoms of this disease. With other prevalent diseases, fasting has included performing a typical 168-hour fast and varying fasting schedules. For instance, in some cultures and religions, fasting is performed over an extended period, only eating one meal at the end of the day and fasting until the same time on the next day. This is done during a popular holiday called Ramadan, which has been scientifically researched for Alzheimer's patients as a possible relief from many of the cognitive symptoms. There have been many promising results when looking at these patients after administering only one meal after the sun goes down. This is seen by improving mitochondrial health and DNA repair, overall glucose homeostasis, generation of ketones, and mobilization of fatty acids [3]. Neuronal plasticity in the hippocampus plays a crucial role in memory and learning; this area is also vulnerable to degeneration and dysfunction in strokes, MS, Alzheimer’s, dementia, traumatic brain injury, epilepsy, and chronic traumatic encephalopathy.

**Figure 1:**
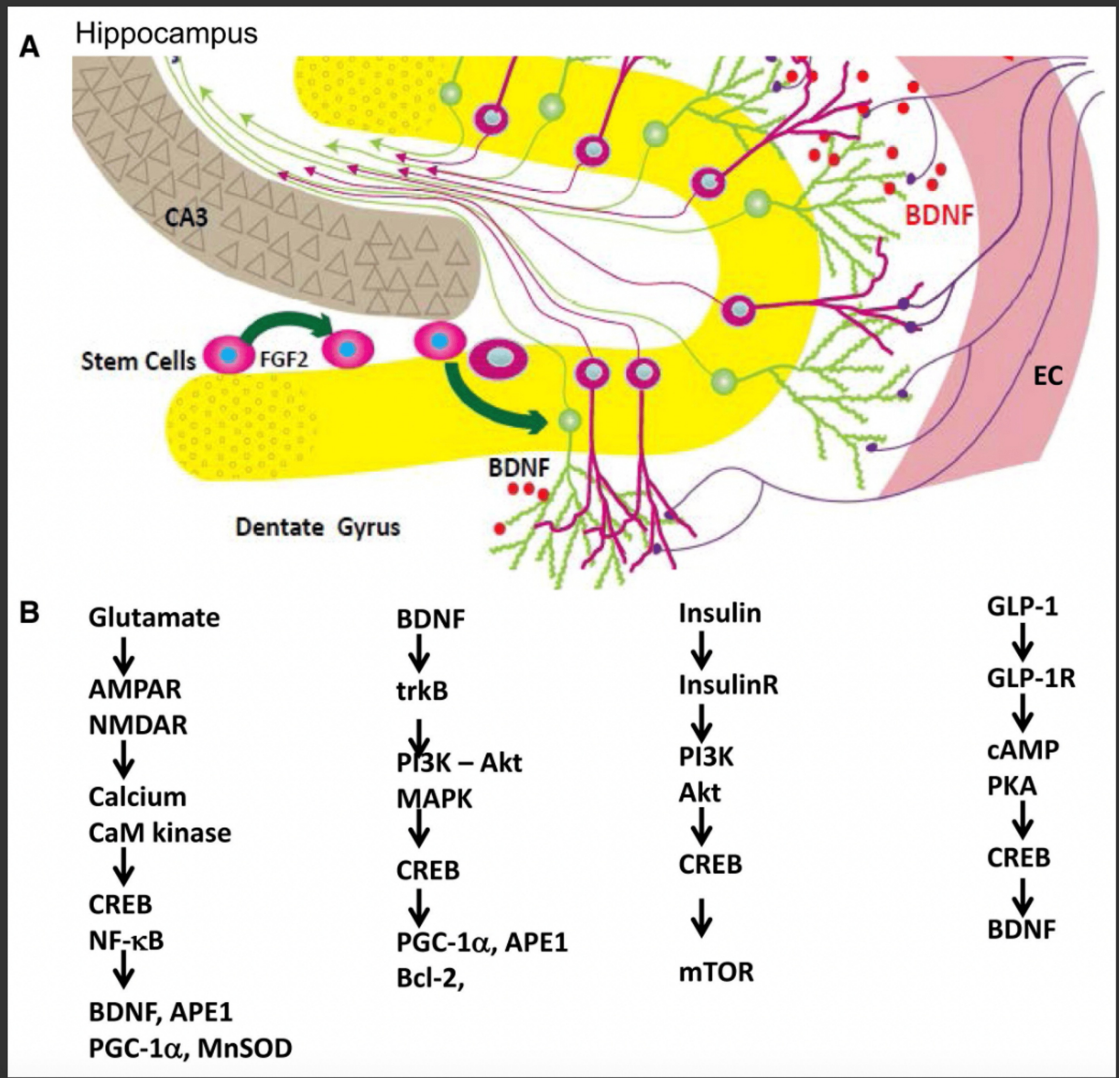
Inflammatory pathways upregulated in the hippocampus during MS and the secondary messengers involved. A: Neurons in the dentate gyrus (yellow) receive inputs from the entorhinal cortex (EC), which acts as a conduit for sensory information originating from higher cerebral cortical regions involved in processing sensory inputs and internally generated cognitive functions. Neurons in the hippocampus are crucial for learning and memory and are particularly vulnerable to dysfunction and degeneration in conditions such as Alzheimer’s disease, stroke, traumatic brain injury, and epilepsy. B: Glutamate, BDNF, insulin, and glucagon-like peptide 1 (GLP-1) activate signaling pathways that enhance neuronal bioenergetics and protect neurons from neurodegenerative diseases and traumatic injury. BDNF: Brain-derived neurotrophic factor; mTOR: mammalian target of rapamycin; MS: multiple sclerosis Source: Reference [8]. Permission was obtained from the original authors for this image

Figure 1 shows a summary of the intracellular pathways activated when your body turns to a fasting state from a fed state. Again, this is not a comprehensive, detailed list of every single path that is upregulated or downregulated in the body, but just a broad overview of the main factors involved in our body's response to a caloric deficit. Also seen is a visual representation of the dentate gyrus's role in the proliferation and differentiation of hippocampal neurons. Again, the formation of these neurons is critical to the formation and consolidation of new memories, which seem to be significantly injured in response to the demyelination of the axons seen in MS. Adiponectin substantially increases in response to fasting; data suggest roles for adiponectin in the alleviating effects of IF on the cardiovascular system [6]. The hunger response from the hypothalamus may also increase immune function during aging, as ghrelin-deficient mice would display increased thymic involution [6]. Another essential factor that will be looked at later in this paper is lesion activity because it correlates to increased T-cells seen within the tissue [7]. Chronically active lesions have a broad rim of macrophages with myelin degradation products at the edge [7].

## Review

Different forms of IF for mice and homo sapiens

IF is often perceived by the general public as a form of extreme restriction or as being synonymous with “starving oneself,” which may contribute to misconceptions. However, the scientific use of IF encompasses structured dietary approaches that should be described in detail. Three popular and widely adopted fasting protocols are used today: alternate-day fasting, whole-day fasting, and time-restricted eating [3]. Alternate day feeding is switched off between ad-libitum (grazing period) and fasting days, which includes almost a quarter of dietary caloric intake. Time-restricted eating is the most popular of the three because of its relative ease of adoption without completely throwing off your metabolic balance. Time-restricted eating is also what most people think of when they hear IF because it follows a rigorous schedule where each day has an eight or 10-hour feeding window during the same time frame each day, without any days skipped [3]. Lastly, whole-day fasting is the most difficult of the three to sustain because of its unorthodox nature. In this method, an individual has to fast for at least one day without food or drink except water. IF and fasting are necessary to distinguish from one another, as fasting involves the complete absence of food. In contrast, IF is a time-restricted habit with calorie intake carefully measured. IF can also include eating low calories for a certain number of days and then returning to the average recommended intake of calories for the rest of the days [5]. IF can take on many forms, mainly changing with calorie intake and feeding time. When these two independent variables are manipulated, one can adhere to a unique IF schedule. In a study done by Wingo et al., there were indeed several limitations to utilizing the human model due to a lack of proper dietary adherence. Participants reported adhering to the IF regimen on average ≥6.5 days per week by the midpoint and final week of the study. However, analysis of three-day, 24-hour food recall data indicated that actual meal timing did not always align with the prescribed fasting schedule, suggesting that participants may have overestimated their adherence [9].

In mouse model studies, however, IF and time-restricted eating have shown auspicious results regarding the alleviation of cognitive impairment and increased mental acuity across the board [10]. Critical cellular processes such as beta-oxidation were found to be upregulated within the glycolytic muscles of intermittently fasting mice; essentially, these mice became fat-burning machines [10]. There was a decrease in lipid droplet formation in the white adipose tissue of these IF mice and better insulin resistance, as seen in HOMA-IR levels, a measuring tool for insulin levels within the blood. The prominent finding within this study was that IF and calorie restriction led to the amelioration of T2DM in New Zealand Obese (NZO) mice, a mouse model representing obesity and T2DM [11]. Preclinical and limited clinical studies suggest that IF and calorie restriction may provide potential benefits for metabolism-related pathologies and neurocognitive conditions. These findings will be discussed further in this review. While numerous mouse model studies support the benefits of IF, evidence in humans remains limited, particularly for MS and other cognitive diseases. Therefore, the proposed experiment focuses on the EAE mouse model, as conducting human trials would require substantial funding and raise ethical challenges.

Specific aims

The primary aim of this study is to assess whether prolonged IF may have beneficial effects on hallmark symptoms associated with MS in the experimental autoimmune EAE mouse model. These symptoms include cognitive, motor, sensory, and other neurological impairments. This study is exploratory in nature and intended to generate preclinical evidence to guide future investigations in humans.

IF is a time-restricted eating schedule where the body's metabolism is downregulated for an extended period, typically 14 to 18 hours. This method of feeding has gained popularity in modern society because of its potential for weight loss and longevity [1]. It is imperative to discover if there is a direct correlation between IF and the reduction of cognitive impairment associated with MS because many of the pharmacotherapies provided today come with a wide range of side effects and unforeseen consequences. A time-controlled diet would have a less severe impact on MS patients and provide a lifelong alternative to expensive and dangerous drug treatments. It is essential to note that conducting a study that looks at all the symptoms of this degenerative, autoimmune disease is extremely difficult due to the vast and diverse variety of symptoms MS patients experience. Numerous analytical methods for cognitive assessment in MS patients are used today, which include the Brief International Cognitive Assessment for Multiple Sclerosis. The brief international cognitive assessment is a cognitive test that’s used in clinical research and practice, which includes the Symbol Digit Modalities Test (SDMT), the Brief Visuospatial Memory Test-Revised (BVMT-R), and the Rey Auditory Verbal Learning Test (RAVLT) [12]. These specifically test the visuospatial and auditory ability of MS candidates. However, there needs to be a benchmark cognitive test that matches specific criteria to indicate the severity of the disease before and after treatment. Because we are explicitly testing on EAE mice models, other objectives that would be interesting to look at relating to MS pathologies include the following:

In addition to proper adherence to IF, intake of specific food groups during non-fasting periods should be studied across the board via ratios of carbohydrates, fats, and proteins. For example, research could further assess the breakdown of micronutrients concerning complex vs. non-complex carbs, saturated fats, unsaturated fats, lean protein vs. animal protein, etc. This variable of food choice could introduce confounding factors in our results because, as we know, different foods are processed differently by each individual based on varying metabolisms, based on criteria such as weight, fat metabolism, energy expenditure, and weight changes. It is also important to note that mice and humans differ significantly in their metabolism, which limits the direct generalizability of findings from the EAE model to human MS outcomes. Recognizing this distinction reinforces the preclinical scope of the present study.

As an exploratory preclinical study, this work may generate evidence suggesting that IF could potentially delay or alleviate MS-like symptoms in the EAE mouse model. Such findings would not establish clinical conclusions but could provide valuable insight into mechanisms affecting cognitive dysfunction, motor control, and muscle weakness, thereby supporting the rationale for future investigation in humans.

To fully understand the effect of IF on patients with MS, it is critical to look at what is happening intracellularly. To do this, the proposed study will look at specific transcription factors that are upregulated and/or downregulated after placing the MS patients on a strict, timed diet. Furthermore, specific secondary messenger cascades and signaling pathways will be closely observed before, during, and after the study. Specific pro-inflammatory cytokine levels, such as interleukin-1 (IL-1), IL-6, IL-12, and tumor necrosis factor-alpha (TNF-α), will be measured. Studies in the past have shown that there is a possibility that inflammatory pathways are activated independently of neurodegenerative-associated MS, while others have shown that the two are interrelated [3]. A secondary goal of the study is to interpret the relationship of inflammation, neurodegeneration, and disease advancement in different MS stages concerning lesion activity, with a specific focus on progressive MS. In addition to looking at the particular cytokines above, a lymphocyte proliferative assay will be utilized to evaluate the immune system for all of the EAE model mice, specifically, cell-mediated immunity. Cells undergoing proliferation elevate their rate of DNA and protein synthesis, and this increase in DNA synthesis can be evaluated by introducing [3H] thymidine, a radioisotope-labelled precursor to DNA [3].

Three main transcription factors, FOXP3, SOX30, and NFAT5, will be examined to determine the specific intracellular signaling. These three transcription factors have been implicated in activating pro-inflammatory cytokines within MS candidates [13]. The upregulation or downregulation measured by the expression levels will give insight into how different pathways are turned on and off in response to IF in the mice.

Does IF Mimic Caloric Restriction, or Are There Two Separate Activated Pathways?

If it can be deduced that calorie restriction and IF show similar outcomes, most likely identical pathways are activated within the mouse model. This can eventually lead to more future research where calorie restriction and IF are adopted in the same mouse model to observe if the effects are amplified and if this is a safe practice method for MS patients. This study’s approach to answering these questions will start with necessary and insightful background research and try to discover how MS is connected to caloric restriction and IF.

Intermittent fasting and multiple sclerosis

**Figure 2:**
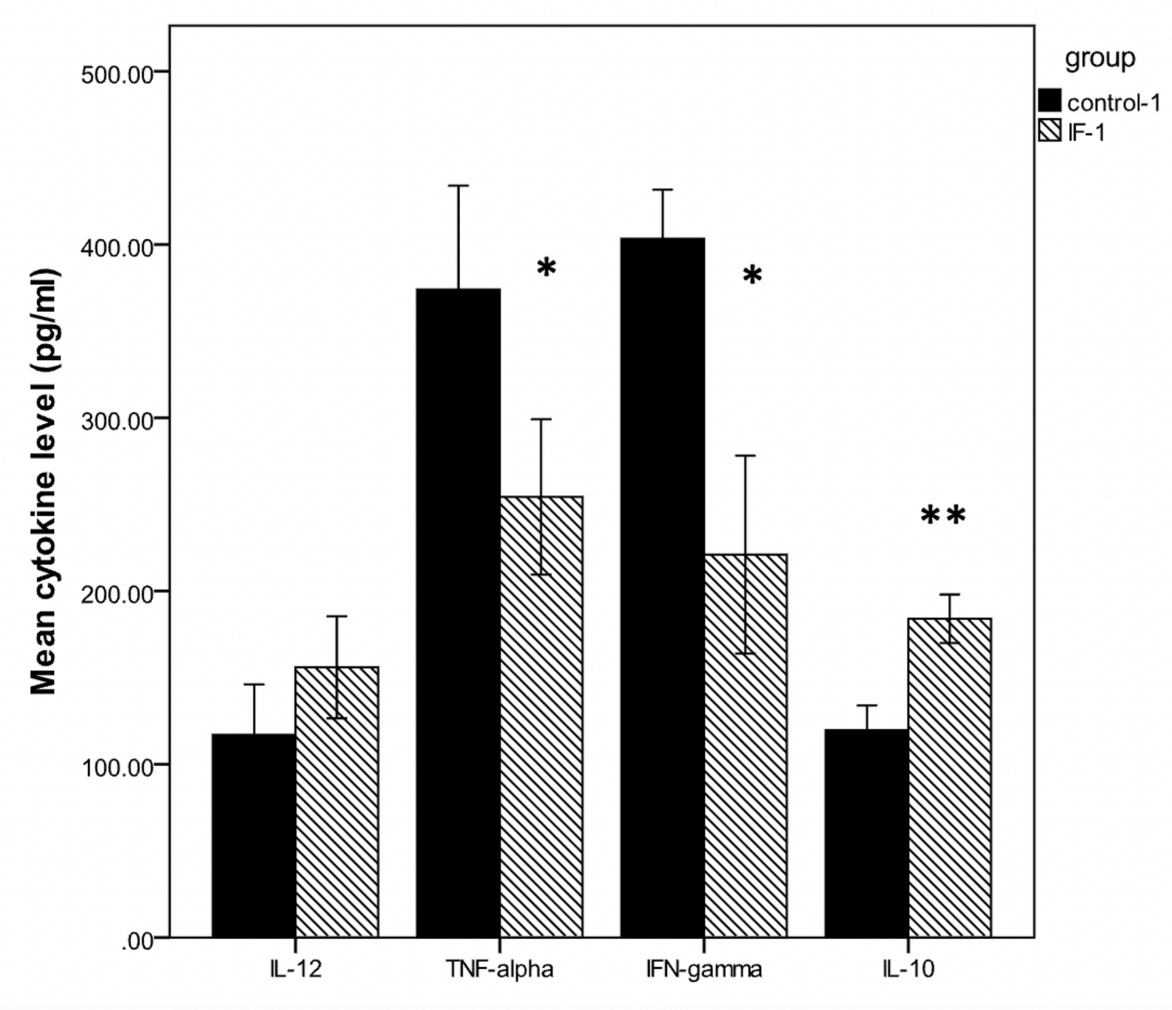
Cytokine levels were measured in MS-induced C57BL/6 mice for the intermittent fasting control group. This data was acquired by adopting cytokine content in supernatant and ELISA kit quantification. MS: Multiple sclerosis; TNF-alpha: tumor necrosis factor-alpha; IL-12: interleukin-12; IL-10: interleukin-10 Source: Ref [14]. Permission was obtained from the original authors for this image

From previous research, we understand that fasting decreases insulin levels, aids in cellular repair, elevates human growth hormones, and helps regulate genes that mediate longevity and protection from disease [3]. In mouse models, it has been shown that IF has the potential to delay the onset of the disease, lessen disease severity, and reduce the marked inflammation and negative hormonal changes associated with MS. Even though multiple animal studies have displayed the protective mechanism of fasting in MS, results and data on the effects of IF on the outcomes of patients have been scarce. IF after the establishment of EAE had no unfavorable impact on disease development. In addition, fasting at the early stages of the disease alleviated EAE severity by mitigating spinal cord demyelination [14]. Fasting decreased IFN-γ and TNF-α secretion and elevated IL-10 production in splenocytes. Fasting was also associated with decreased migration of pro-inflammatory cytokines such as IL-1, IL-6, and IL-12 to local sites of inflammation [14]. This is shown in Figure 2, where the researchers looked at all four pro-inflammatory cytokine levels measured by looking at the supernatant content under ELISA kits [14].

Another essential aspect to consider for this study is how the human body usually responds under fasting conditions, because there are multiple similarities between regular caloric restriction/typical fasting and IF. Under normal fasting conditions, IF has been proven to decrease resting insulin levels and shift your body's metabolism to breaking down fats for energy. In addition, the human body begins to promote cellular pathways involved in stress resistance, lipolysis, and anti-aging effects [8]. It has even been shown that fasting will reduce mean body weight, increase insulin sensitivity, and reduce inflammation [8]. Considering all these claims is essential because it can help connect the critical points while interpreting the results. Even though different animal studies have displayed the protective mechanism of fasting on MS patients, data and results on the effects of IF on the outcomes of patients have been scarce.

Exploring IF effects on MS with animal models

To answer the aims of this research proposal, a specific experimental design needs to be appropriately carried out. For the primary objective of determining if prolonged IF (> 3 months) relieves any hallmark symptoms of MS, a randomized controlled animal experiment is best suited, as there needs to be extensive time to evaluate metabolic changes in the EAE mouse model. The EAE mouse model is popular in scientific studies today to measure MS pathologies in human and mouse models. The process for generating this mouse model from scratch is described below.

A total of 20 pathogen-free C57BL/6 mice will be used for this experiment. The sample size was determined based on a power calculation (α=0.05, 80% power) and effect sizes reported in prior EAE studies (Cohen’s d ≈ 1.0-1.2), which indicate that 11-13 mice per group are typically sufficient to detect meaningful differences in cytokine expression and clinical scoring. To allow for potential variability in disease induction and attrition, we conservatively selected 20 mice. This ensures adequate statistical power while adhering to ethical principles of minimizing unnecessary animal use. Then, active immunization will be used to induce MS demyelinating symptoms and neuroinflammation. This active immunization works by injecting a toxin, in most cases, pertussis toxin, to purposely elicit an immune response from the mice.

Injection of pertussis toxin induces MS-like symptoms via the induction of the proinflammatory cascade of TGF-β, IL-6, and Th17 in the CNS, which has been seen as essential in the development of the EAE mice model [15]. The mice will then have an overabundance of cytokines migrating throughout the blood, often termed cytokine storm; this is a result of a superantigen response from MH2 on an antigen-presenting cell binding to a CD4 molecule on a B-cell. Finally, this will induce sufficient inflammation throughout the brain and cause severe lesions that mimic some of the lesions and enlarged ventricles in human subjects. In addition, the brains of the mice should have atrophied, as shown by decreased mass and an increase in sulci width and depth.

Parameters for this study include: the mice must adhere to a rigorous eating schedule, and the mice need to be immune to secondary diseases (verified via screening) such as T2DM, hypertension, immunodeficiency/autoimmune diseases, or any condition that would affect the results of this study. After the 20 randomly selected MS-induced mice are chosen, the next step is administering or outlining a diet for the non-fasting hours. Next, they will be split into two groups, with ten mice following the 16:8 fasting method and the other ten following the 14:10 fasting method, to see if there are significant differences between them. For the methods of the experiment, immunohistochemistry will be utilized to see if there is proper migration in the dentate gyrus from the marginal zone to the submarginal zone, where there is sufficient neuronal proliferation. A lack of migration or proliferation of hippocampal neurons in the dentate gyrus can contribute mainly to the symptoms seen in MS.

**Figure 3:**
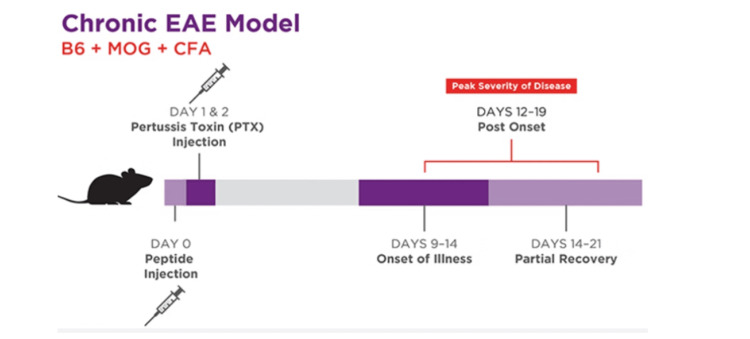
Chronic EAE mice model that will be used to induce MS and the timeline of pertussis injection EAE: Encephalomyelitis; MS: multiple sclerosis Source: Ref [18]. Permission was obtained from the original authors for this image

Before experimentation, data relating to each mouse’s body weight, baseline inflammation measured by cytokine levels and C-reactive protein, and fasting insulin levels in the blood will be recorded. LDL-C, triglycerides, and blood pressure will also be measured. From previous research, these are adequate parameters to measure overall metabolic and mitochondrial dysfunction [16]. Before manipulating the mice's diet, which will be approximately given 60 percent carbohydrates, 30 percent protein, and 30 percent fat [17]. This distribution of macronutrients has been previously shown to reduce autoinflammation in experimental mouse models [17]. Next, similar conditions will be used on previous mouse models, such as infiltrating pro-inflammatory immune cells into the CNS. We can develop our mouse model using two methods: active immunization for T-cells that specifically target the myelin sheath or passive induction with the addition of active transfer. In this experiment, active immunization will be adopted where mice will be injected with CNS-specific antigens, which elicit the formation of myelin-specific T-cells, leading to the mobilization of pro-inflammatory cytokines and direct damage in the CNS [6]. This method is chosen because it is inexpensive, quick, and efficient. To look at the specific intracellular pathways upregulated following an IF diet for the model mice, we will precisely measure the expression levels of transcription factors to see if there is a significant difference from baseline levels. Figure 3 displays how the EAE model of mice is generated using the active immunization technique. On the first day, pertussis toxin injections are carried out to stimulate the T-cell response of the mice, and then, eventually, there will be a partial recovery. The mice's immune system typically takes around 9-14 days to respond to the injection, by which point there is post-onset and eventual partial recovery from the mice.

After generating the MS mouse model, the next step is to fast the eight-month-old mice for 24 hours and have them injected with cytokine markers that measure pro-inflammatory cytokines. A radiolabeled dye such as fludeoxyglucose (18F) will measure glucose utilization in the prefrontal cortex and the hippocampus because these are the two areas of the brain that are impacted by MS the most [19]. The hippocampus mainly controls the consolidation of memories, and the prefrontal cortex deals with executive decision-making and overall cognitive function. It is important to note that our vehicle mice will also have MS-like symptoms via the EAE model, except there will be no diet manipulation, and they will choose when to graze freely. Next, we will measure fasting glucose levels, insulin levels in the blood, and migratory cytokine levels for all the mice. These endpoints are crucial to measure. It has been shown that IF and caloric restriction have been shown to significantly benefit T2DM patients [19]. After all the baseline levels have been recorded at the experiment's inception, the next step is to start the IF process in our mouse model, which will continue for six months.

To measure the expression levels of the chosen transcription factors, we will generate a new transgenic mouse model with the KO of SOX30 while also looking at inflammatory responses within these mice after IF (two mice). This will be done using CRISPR-Cas9 gene editing, a tool used in gene therapy, where certain genetic diseases, such as MS, can be potentially alleviated. Gene therapy will allow for the insertion of a particular vector carrying a gene of choice into the genome of the recipient. This therapy can replace a damaged gene or restore the total function of the protein. Specific TF’s like FOXP3, SOX30, and NFAT5 will be studied using an SDS-PAGE. These transcription factors have been previously implicated in progressive MS in mouse models as having significant upregulation and are thought to play a significant role in secondary inflammatory processes. At the end of every week, glucose and insulin levels will be measured using HOMA-IR. Inflammatory cytokine levels, specifically IL-1 and IL-6, will be measured in the same time frame to see if there are significant inflammation levels as shown by elevated cytokine levels in blood. In the brain, using F18 radiolabeling and confocal microscopy, we can quantify levels of marked inflammation within the prefrontal cortex and hippocampus.

At the end of the IF period, data will be collected from all enrolled mice for each parameter (glucose/insulin levels, inflammatory cytokines, and expression levels of SOX30/FOXP3). These measurements are expected to yield 20 data points per group, corresponding to the planned sample size. This reflects the study design rather than the results obtained. Statistical analysis will include one-way ANOVA and t-tests to observe if there is a significant difference between the control mice and the experimental mice.

**Figure 4:**
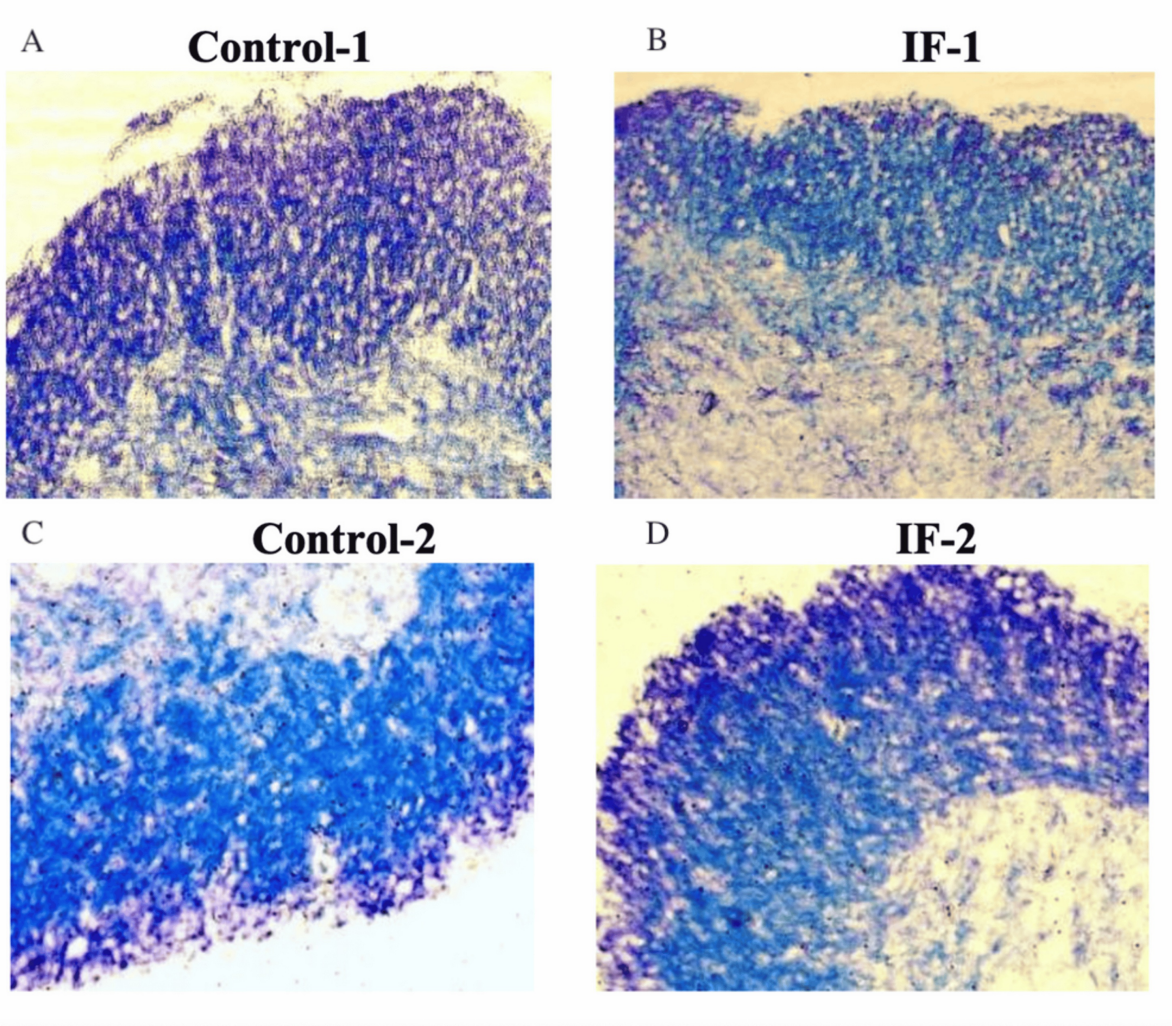
Immunohistochemistry results from Cresyl Violet staining IF-1: Intermittent fasting after appearance of the first clinical sign for 10 days, IF-2: Intermittent fasting 30 days post immunization for 10 days, Control-1: normal diet for 21 days, Control-2: normal diet for 40 days Source: Ref [14]. Permission was obtained from the original authors for this image

Another experimental measure to be looked at is the demyelination of the spinal cord itself in mice that is induced by activated immunization (EAE). The adopted method is immunohistochemistry and uses LUXOL Fast Blue and Cresyl violet staining. In this stain, the images will have a distinct blue and purple pattern: purple represents the areas of demyelination, and blue represents the still intact myelin. From these images, the proportion of demyelination using the ratio of blue area to purple pattern will be quantified and plotted on a bar graph for each mouse after the three months have concluded. Figure 4 shows the expected results that will come from performing the immunohistochemistry [14].

Inflammation as a catalyst for MS disease progression

Inflammation is a key driver in the pathogenesis of MS, contributing to both demyelination and neurodegeneration. MS is primarily mediated by autoreactive T cells, particularly Th1 and Th17 subsets, which infiltrate the CNS after breaching the blood-brain barrier, leading to an inflammatory cascade [20]. Activated microglia and macrophages release proinflammatory cytokines such as TNF-α and IL-17, which exacerbate myelin and axonal damage [21]. Additionally, B cells contribute to MS pathology by producing autoantibodies and acting as antigen-presenting cells, further amplifying T cell-mediated immune responses [22]. Chronic neuroinflammation also involves oxidative stress and mitochondrial dysfunction, leading to progressive neurodegeneration [23]. Although regulatory T cells (Tregs) typically suppress excessive immune activation, their dysfunction in MS promotes sustained inflammation and disease progression [24].

Understanding these inflammatory mechanisms has led to the development of disease-modifying therapies that target immune responses, aiming to slow disease progression and mitigate neuronal damage [20].

Emerging research suggests that dietary interventions, such as IF, may help modulate inflammation in MS by altering immune responses and metabolic pathways. IF has been shown to reduce systemic inflammation and promote a more anti-inflammatory immune profile, which could be beneficial in MS management [25]. Additionally, obesity is a known risk factor for MS, as excess adiposity contributes to chronic low-grade inflammation and immune dysregulation, further exacerbating disease progression [26]. While disease-modifying therapies remain the cornerstone of MS treatment, they can have significant side effects, including immune suppression and metabolic disturbances, highlighting the need for complementary approaches like dietary interventions [27]. By improving metabolic resilience and reducing proinflammatory cytokine production, dietary strategies such as IF may serve as an adjunct therapy to mitigate MS progression and treatment-related complications [28].

The persistence of inflammation in MS is driven not only by autoreactive T and B cells but also by dysregulated innate immune responses. Microglia and infiltrating macrophages become chronically activated in the MS lesion environment, releasing proinflammatory mediators such as TNF-α, reactive oxygen species (ROS), and nitric oxide, which amplify neurotoxicity and contribute to ongoing demyelination [29]. This chronic inflammatory milieu fosters a feed-forward loop in which immune cells perpetuate damage, impair remyelination, and accelerate neurodegeneration. While disease-modifying therapies (DMTs) effectively reduce peripheral immune activation, their impact on CNS-resident inflammation remains limited, underscoring the need for alternative approaches that target neuroinflammation more directly [30]. Emerging evidence suggests that metabolic interventions such as IF may help suppress CNS inflammation by modulating energy metabolism and immune cell function. IF has been shown to reduce microglial activation and shift macrophage polarization toward an anti-inflammatory phenotype, thereby dampening neuroinflammation [31]. Additionally, fasting-induced changes in ketone body production can enhance neuronal resilience and promote a less inflammatory CNS environment, potentially counteracting some of the neurodegenerative processes seen in progressive MS [32]. By targeting both peripheral and CNS inflammation, metabolic interventions may serve as a promising adjunct to traditional therapies, offering a novel strategy to mitigate the long-term inflammatory burden in MS. B cells further contribute to the inflammatory burden in MS through antigen presentation and cytokine release, with evidence supporting that B cell depletion therapy not only decreases relapses but also attenuates CNS-compartmentalized inflammation, pinpointing its role beyond antibody production [8,33] in mouse models.

Future implications of the study

The results of this study point to the significance of IF on MS pathologies. Suppose it is concluded that there is a significant difference; in that case, future questions should be asked. If this method of IF translates to human models, and similar intracellular pathways will be affected, can this therapy be generalized to most individuals affected by MS? Also, suppose there is a marked increase or decrease in the frequency and size of brain lesions measured by the fludeoxyglucose. What does this mean for future experimentation using EAE mouse models using IF protocols? For the last aim, if calorie restriction mimics IF, previous research will be compared to the research gathered from experimentation. Data will be cross-matched to see if it can be hypothesized that calorie restriction mimics IF. If this is the case, many of the proven benefits provided by caloric restriction from prior studies, such as increased cognitive function and anti-aging effects, are also produced from a strict IF diet [17]. This, if verified by a clinical trial, could potentially provide support for physicians and caregivers to prescribe this regimen based on its beneficial impact on the human body. Potential limitations of this study are that only some cytokines, which may not fully represent all inflammatory or signaling pathways relevant to MS, are being measured, and only some pertinent transcription factors are being examined. Suppose we were looking at the broad spectrum of cytokine profiles. In that case, we can conclusively see all marked inflammation without assuming that other cytokine levels would be elevated, similar to Figure 4. Also, another potential limiting factor is that some of the mice can respond differently to IF, and some mice, when using active immunization, can develop secondary symptoms of MS. In addition, levels of glucose and insulin could be extremely elevated in the case that one or more of the mice have a hypersensitivity reaction. This hypersensitivity reaction can range from a simple secondary reaction to an elimination or reduction of certain foods, or it could extend all the way to an unpredictable response in the immune system, such as T-cell overactivation, which can result in organ failure and respiratory failure. It is imperative to note that although the results may show a correlation between IF and MS, more studies have to be conducted to help support this hypothesis.

It should also be noted that in previous studies, it has been shown that sometimes when first following the IF routine, there is a significant improvement in the MS patient’s symptoms, but then eventually falls back to baseline; this means that there is another possible time component that needs to be accounted for when interpreting results. If there is a considerable reduction in cytokines and overall inflammation after just one week of abiding by the IF diet, it can be a result of the body getting adjusted to a new lifestyle. The results midway and towards the end of the study are more important indicators as to how the mice are adjusting to IF, compared to the first couple of hours or days.

## Conclusions

MS is a prevalent neurodegenerative autoimmune disorder for which current therapies are predominantly pharmacological, with varying levels of effectiveness and a number of serious side effects. IF has been linked to improved weight regulation, reduced systemic inflammation, and favorable metabolic effects in both experimental and clinical settings. These findings provide a rationale for considering IF as a potential non-pharmacological strategy for investigation in MS; however, the current evidence remains preclinical, and there is no direct clinical evidence in MS patients to date. The experimental autoimmune EAE mouse model provides a safe and reproducible means to study the effects of IF without involving human participants. While this model allows mechanistic insights, important physiological differences between mice and humans limit direct translation, underscoring the need for validation in clinical trials. Research in this area remains hypothesis-generating but is promising for understanding inflammatory processes in MS and other autoimmune or neurodegenerative diseases.

Because IF has been associated with reductions in systemic inflammation, it represents a promising, cost-effective, non-pharmacological approach worth exploring in the EAE model of MS. However, its applicability to human MS remains unproven, and clinical studies will be required to establish safety and efficacy. To advance toward clinical relevance, future investigations must demonstrate consistent reductions in pro-inflammatory cytokines, measurable improvements in demyelination by histopathology, stabilization of glucose-insulin homeostasis, and the absence of adverse immune reactions. Until such criteria are met in well-designed human studies, the role of IF in MS should be regarded as preliminary and exploratory
